# The human metabolic profile reflects macro- and micronutrient intake distinctly according to fasting time

**DOI:** 10.1038/s41598-018-30764-4

**Published:** 2018-08-16

**Authors:** A. Sedlmeier, A. Kluttig, I. Giegling, C. Prehn, J. Adamski, G. Kastenmüller, M. E. Lacruz

**Affiliations:** 1 Institute of Medical Epidemiology, Biostatistics and Informatics, Martin-Luther-University Halle-Wittenberg, Halle, Germany; 2 Department of Epidemiology and Preventive Medicine, University of Regensburg, Regensburg, Germany; 3 Clinic of Psychiatry, Psychotherapy, and Psychosomatic, Martin-Luther-University Halle-Wittenberg, Halle, Germany; 4 Institute of Experimental Genetics, Genome Analysis Center, Helmholtz Zentrum München, Neuherberg, Germany; 5 Institute of Experimental Genetics, Technical University Munich, Freising-Weihenstephan, Germany; 6 German Center for Diabetes Research (DZD), Neuherberg, Germany; 7 Institute of Bioinformatics and Systems Biology, Helmholtz Zentrum München, Neuherberg, Germany; 8 Department of Twin Research and Genetic Epidemiology, King’s College London, London, UK

## Abstract

Although the impact of dietary patterns on human serum metabolites has been examined, the fasting effect on the metabolic profile has not yet been considered. The aim of this cross-sectional study is to investigate the influence of fasting regarding the association between dietary patterns, reflected by macro- and micronutrient intake, and human serum metabolites in a population-based cohort. A total 1197 non-diabetic German adults aged 45 to 83 years, who participated in baseline of the CARLA study 2002–2006 and had metabolite quantification were selected for this study. Macro- and micronutrient intakes were estimated from a food frequency questionnaire (FFQ). Concentrations of 134 serum metabolites were measured by targeted metabolomics AbsoluteIDQ p150 Kit. The association of dietary patterns with serum metabolites was calculated by means of linear regression and the influence of the fasting status was considered by including interaction terms with each macro- and micronutrient. Higher self-reported intake of alcohol and lower self-reported intake of organic acids were associated with higher concentrations of acylcarnitines and phosphatidylcholines. Mainly the associations between dietary patterns and acylcarnitines and hexose were altered after including interaction terms, suggesting effect modification by fasting status. No effect from fasting time was seen for amino acids and saturated, mono- and polyunsaturated phosphatidylcholines.

## Introduction

Human health is a complex interaction of genetic predisposition and environmental factors^1^. Nutrition is a modifiable risk factor for chronic disease^2^ and one of the most influential environmental factors over the life course^3^. Dietary patterns of individual foods, recorded in a food frequency questionnaire, are an effective approach to examining the relationship between diet and the risk of chronic diseases^4^. The analysis of dietary patterns considers the effects of overall diet and has a broader representation of nutrient consumption. Thus, it has been found that specific dietary patterns, more than individual foods, are predictors of morbidity^5^ and mortality.

Metabolomics is the study of small molecules (metabolites) of an organism at a given time point^6,7^. Those metabolites reflect not only genetic components but also the influence of environmental factors (i.e. drugs, toxins, micro biotic activity of the gut or nutrition)^8^.

Several epidemiological studies have used a metabolomics approach to investigate the effect of nutrition on the metabolic state. A study with data from the EPIC (European Prospective Investigation into Cancer and Nutrition) cohort with 2380 participants investigated the association of food groups assessed by a food frequency questionnaire (FFQ) and the metabolic profile^9^. They reported that nutrition has only a subordinated role on the metabolite profile variation. The TwinsUK-study examined also the association between dietary patterns, determined by factor analysis and metabolic profile^10^. They found that especially coffee, fruit and vegetable consumption were associated with concentrations of acylcarnitines, glycerophospholipids and sphingomyelins. Finally a study with data from the KORA (Cooperative Health Research in the Augsburg Region) cohort investigated how self-reported consumptions of different food groups (18 items) were related to concentrations of metabolites in blood^11^. A clear association was found between self-reported nutrition habits and differences in human metabolic profiles and concluded mainly concentrations of subgroups of phosphatidylcholines reflected the self-reported nutritional intake.

While some studies showed an effect of dietary patterns on serum metabolites^10,11^, others reported a lack of association^9^, leading to contradictory results. This contradiction can be partially due to differences in the conceptualization of nutrition, but also in the statistical analysis. Furthermore, those previous studies did not consider the impact of the fasting status on the metabolic profile. Thus, the objective of the present study is to investigate whether there is an association between dietary patterns, represented by macro- and micronutrient intake, and serum metabolites in participants of the CARLA-cohort. Furthermore the moderator effect of fasting status will be examined, because the postprandial status can greatly influence concentrations of metabolites in blood^12^.

## Methods

### Study population

**Figure 1:**
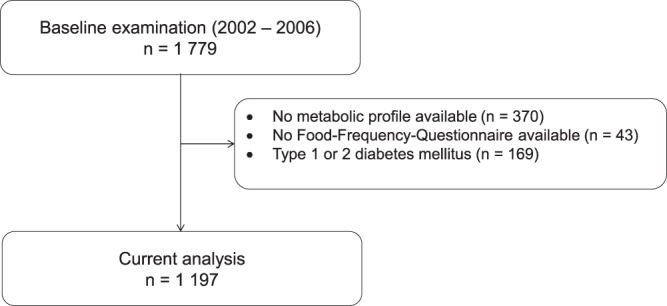
Flow chart of the study population.

The CARLA-Study is a population-based cohort in an elderly population of the city of Halle/Saale in eastern Germany. Study design and methods were described in detail elsewhere^13,14^. In brief, subjects were recruited randomly from the population registry in a multi-stage process. At baseline, 1779 subjects (46% women) aged 45 to 83 years were examined between July 2002 and January 2006 with a response rate of 64%. The current analysis included a total of 1197 participants, who were free of diabetes mellitus (defined by self-report and/or medication), had metabolite measurements and completed a food frequency questionnaire (Fig. 1). The CARLA study was carried out in accordance with the declaration of Helsinki. All participants gave their written informed consent. The study was approved by the local ethics commission of the Medical Faculty of the Martin-Luther-University Halle-Wittenberg.

### Metabolomics measurements

For this study, blood serum samples of the study participants were analysed using a targeted metabolomics approach. Blood collections were spread throughout the day 8 am to 8 pm; approximately 40% of the samples were collected between 8 and 12 am; and 45% between 1 and 4 pm. Blood samples were taken after a supine rest of 30 minutes. After a 10-min centrifugation (20 °C, 1500 RPM), the serum was collected and after a clotting time of 30 minutes, deep frozen to −80 °C on the same day and stored until analysis of the metabolites.

Metabolite quantification was performed in the Genome Analysis Center at the Helmholtz Zentrum München. Out of 10 µL blood serum we quantified simultaneously a panel of 163 metabolites that included free carnitine, 40 acylcarnitines (acylC), 14 amino acids (AA), hexoses (sum of hexoses), 92 glycerophospholipids (15 lyso-phosphatidylcholines (lysoPC) and 77 phosphatidylcholines (PC)), and 15 sphingomyelins (SM) using flow injection analysis-tandem mass spectrometry (FIA-MS/MS) and the Absolute*IDQ* p150 Kit (Biocrates Life Sciences AG, Innsbruck, Austria). The assay was performed on a double-filter 96 well plate containing isotope-labelled internal standards which were taken as reference for metabolite quantification. The procedures for sample preparation and mass spectrometric measurements as well as the metabolite nomenclature have been described in detail previously^15^. The method has been successfully applied in multiple academic and industrial settings. For a full-list of all quality-controlled metabolites, see Supplementary Table 1. Two metabolites (lysoPC a C6:0 and PC ae C38:1) were excluded as the number of missing values within lab analysis exceeded 5% (values = 0). The remaining missing values (1‰ of all values) were imputed using the SAS procedure MI with the MCMC (Markov chain Monte Carlo) method. Imputations were done with minimum and maximum values defined from the CARLA population and every single imputation was plausibility checked. Additionally, 27 (13 acylC, 9 PC and 5 SM) further metabolites were excluded from the analysis as their experimental variation assessed through the coefficient of variation (CV) of 173 measured aliquots of a reference plasma sample (5 on each plate) exceeded 25%. Since blood samples were analysed on thirty-five plates (batches), a so-called batch variable was included in analyses as a random factor in order to avoid possible effects due to technical issues or different time points of analyses. No outliers, defined as greater than mean ± 5 standard deviations of the particular metabolite over the whole population, were found^16^.

To account for differences in chemical structures and physiological function, we created 11 metabolite-subgroups: amino acids were divided in essential or indispensable (Phe, Val, Thr, Trp, Met, xLeu, His) and non-essential or dispensable (Arg, Gln, Gly, Orn, Pro, Ser, Tyr). Acylcarnitines were allocated to short- (C2 to C5), medium- (C8 to C10) or long-chain (C12 to C18) groups. Phosphatidylcholines were separated in saturated PC (PC only with single bonds), monounsaturated (PC with a double bond) or polyunsaturated (PC with two or more double bonds). Sphingolipids, lysoPC and Hexose were considered each as a separate group.

### Dietary pattern

The examinations at baseline investigations included a standardized computer-assisted personal interview, self-administered questionnaires, a medical examination by trained personnel, and drawing of a venous blood sample. The standardized, computer-assisted interview collected information on socio-economic status and life-style variables. Dietary patterns were determined based on the self-administered food frequency questionnaire (FFQ) of the EPIC-cohort^17–20^. Participants were asked how often (on average) they ate during the last year the following 148 food items (e.g. whole meal products, vegetables, chocolate, and meat). The answers could be ranked according to 10 categories. Habitual self-reported macronutrient intake data were derived from the validated self- or interviewer-administered country-specific FFQ or dietary histories taken at baseline, with nutrient composition derived from the EPIC Nutrient DataBase^21^. Nutrients were categorised into the following seven macro- and micronutrient groups (grams per day): alcohol, dietary fiber, protein, fats, carbohydrates, mineral nutrients and organic acids. Macro- and micronutrient groups were log-transformed and standardised to allow comparison among groups^18^.

### Statistical analysis

The statistical analysis system SAS version 9.4 (SAS Institute Inc.; Cary, NC) was used for the statistical analysis. Metabolite groups, alcohol and organic acids were log-transformed since in most cases the log-transformed concentrations were closer to a normal distribution than the untransformed values and normalized to unit standard deviation.

The association of the 7 macro- and micronutrients with the 11 metabolite subgroups (indispensable and dispensable amino acids; short-, medium- and long-chain acylcarnitines; saturated, monounsaturated and polyunsaturated PC; sphingolipids, lysoPC and hexose) was calculated by means of linear regression. We identified minimally sufficient adjustment sets using directed acyclic graphs (DAG) that represent the relations among the exposure, outcome, and other variables^22^. The minimally sufficient adjustment set for the total effect in the association of dietary patterns on metabolites included sex, age, BMI, smoked pack-cigarettes per year and physical activity.

To examine whether the fasting time influences the association between dietary patterns and metabolite patterns, interaction analyses (macro- and micronutrient*fasting time) were performed with *p*-values < 0.1 as a cut-off.

## Results

Table 1 shows the main characteristics of the study population. Only apparently metabolically healthy participants were considered for this study (n = 169 participants with self-reported diabetes or intake of antidiabetic medication were excluded). Thus, the study population comprises 1197 participants, 659 men with a median age of 63 years and 538 women with a median age of 62 years. The mean fasting time was 200 minutes. The mean (SD) concentrations of individual serum metabolites of the population are presented in Supplementary Table 1 and the mean intake of the 7 macro-and micronutrients is presented in Supplementary Table 2.

**Table 1:** Characteristics of the study population (n = 1197).

	Men (n = 659)		Women (n = 538)	
	Median/n	Q3–Q1/%	Median /n	Q3–Q1/%
Age	63.2	71.7–55.0	62.3	68.3–53.8
BMI [kg/m^2^]	27.4	30.0–25.2	27.1	30.3–24.3
Fasting time [Min]	202.0	264.0–153.0	210.0	273.0–158.0
Total cholesterol [mmol/l]	5.3	5.9–4.8	5.7	6.4–5.0
HDL-Cholesterol [mmol/l]	1.2	1.5–1.1	1.6	1.9–1.3
LDL-Cholesterol [mmol/l]	3.2	3.7–2.6	3.4	4.0–2.8
Triglycerides [mmol/l]	1.7	2.5–1.2	1.3	1.8–1.0
Cardiovascular diseases^a^	75	11.4%	23	4.3%
Hypertension^b^	489	74.2%	361	67.1%
Cardiovascular medication (ATCcodes C02/03/07/08/09)	1.0	2.0–0.0	1.0	2.0–0.0
Smoker				
Current	144	21.9%	76	14.1%
Ex	329	50.0%	96	17.8%
Never	186	28.2%	366	68.0%
Pack-years tobacco	27.0	37.1–15.4	19.1	25.7–9.1
Coffee [cups /day]	2.0	4.0–2.0	2.0	3.0–2.0
Tea [cups/day]	0.0	1.0–0.0	0.0	1.0–0.0
Sports-Index (1 = low, 5 = high)	2.3	3.0–2.0	2.3	3.0–1.8

^a^Participants with self-reported diagnosis of heart infarction, CABG (coronary artery bypass graft), PTCA (percutaneous transluminal angioplasty), stroke or Carotis-OP.

^b^Systolic blood pressure ≥140 mmHg or diastolic blood pressure ≥90 mmHg and/or anti-hypertensive medication (ATC codes C02/03/07/08/09).

Beta-estimators for serum metabolite levels from Generalized Linear Models are shown in Table 2. All analyses were adjusted for sex, age, BMI, smoked pack-cigarettes per year and physical activity. None of the beta-estimators of macro- and micronutrients for dispensable amino acids, hexose and sphingomyelins were significant. Dietary intake of organic acids was inversely associated with all types of acylcarnitines and with all groups of phosphatidylcholines. The estimators ranged from −0.14 to −0.08. Alcohol intake was positively associated with saturated, mono- and polyunsaturated phosphatidylcholines. For each of these three beta-estimators the *p*-values were statistically significant, even after correcting for multiple testing with Bonferroni. Alcohol also showed associations with short- and long-chain acylcarnitines and with lyso-phosphatidylcholines. The macronutrient carbohydrate was correlated with medium- and long-chain acylcarnitines with beta-estimators of 0.12. Carbohydrates, as well as fats, were inversely associated with indispensable amino acids.

**Table 2:** Detailed results of the Generalized Linear Model^a^ in the CARLA study – beta estimators for the association between macronutrients (n = 7) and metabolite groups (n = 11), significance level < 0.05.

	Indispensable amino acids (n = 7)			Dispensable amino acids (n = 7)			Short-chain acylcarnitines (n = 5)			Medium-chain acylcarnitines (n = 6)			Long-chain acylcarnitines (n = 8)			Hexose (n = 1)		
	Estimate	SE	*p*	Estimate	SE	*p*	Estimate	SE	*p*	Estimate	SE	*p*	Estimate	SE	*p*	Estimate	SE	*p*
Alcohol	−0.01	0.03	0.74	−0.02	0.03	0.52	**0.09**	**0.03**	**0.01**	0.05	0.03	0.13	**0.10**	**0.03**	<0.0001*	0.05	0.03	0.13
Dietary fiber	0.13	0.06	0.05	0.10	0.06	0.11	−0.04	0.06	0.50	−0.04	0.06	0.53	−0.10	0.06	0.09	0.07	0.06	0.29
Protein	0.08	0.09	0.36	−0.01	0.09	0.91	0.03	0.09	0.71	0.07	0.09	0.39	−0.03	0.09	0.75	0.14	0.09	0.12
Fats	**−0.15**	**0.07**	**0.04**	−0.10	0.07	0.19	−0.11	0.07	0.12	−0.05	0.07	0.50	−0.03	0.07	0.72	−0.12	0.07	0.09
Carbohydrates	**−0.15**	**0.06**	**0.01**	−0.01	0.06	0.91	−0.04	0.06	0.54	**0.12**	**0.06**	**0.04**	**0.12**	**0.06**	**0.05**	−0.08	0.06	0.16
Mineral nutrients	0.13	0.11	0.23	0.02	0.11	0.83	0.14	0.11	0.18	−0.08	0.10	0.45	0.04	0.10	0.72	−0.03	0.11	0.76
Organic acids	−0.06	0.04	0.13	−0.03	0.04	0.40	**−0.13**	**0.04**	<0.0001*	**−0.10**	**0.04**	**0.01**	**−0.14**	**0.04**	<0.0001*	−0.04	0.04	0.37
	**Saturated phosphatidylcholines (n = 12)**			**Monounsaturated phosphatidylcholines (n = 11)**			**Polyunsaturated phosphatidylcholines (n = 49)**			**Sphingomyelins (n = 19)**			**Lyso-phosphatidylcholines (n = 9)**					
	**Estimate**	**SE**	***p***	**Estimate**	**SE**	***p***	**Estimate**	**SE**	***p***	**Estimate**	**SE**	***p***	**Estimate**	**SE**	***p***			
Alcohol	**0.13**	**0.03**	<0.0001*	**0.21**	**0.03**	<0.0001*	**0.14**	**0.03**	<0.0001*	−0.03	0.03	0.33	**0.07**	**0.03**	**0.05**			
Dietary fiber	−0.02	0.06	0.76	**−0.15**	**0.06**	**0.02**	0.02	0.06	0.71	−0.05	0.06	0.40	0.00	0.06	0.97			
Protein	−0.05	0.09	0.58	−0.07	0.09	0.40	0.02	0.09	0.79	0.08	0.09	0.38	0.06	0.09	0.53			
Fats	0.00	0.07	0.95	−0.05	0.07	0.44	−0.05	0.07	0.49	0.01	0.07	0.92	−0.06	0.07	0.43			
Carbohydrates	−0.07	0.06	0.28	0.09	0.06	0.14	−0.03	0.06	0.64	0.06	0.06	0.34	0.02	0.06	0.72			
Mineral nutrients	0.13	0.11	0.22	0.16	0.11	0.14	0.05	0.11	0.66	−0.02	0.11	0.84	0.05	0.11	0.66			
Organic acids	**−0.08**	**0.04**	**0.04**	**−0.10**	**0.04**	**0.01**	**−0.10**	**0.04**	**0.01**	−0.04	0.04	0.26	**−0.11**	**0.04**	<0.0001*			

*p < 0.004 (0.05/11; Bonferroni-corrected).

^a^Adjusted for sex, age, BMI, smoked pack-cigarettes per year and physical activity.

Inclusion of interaction terms (multiplicative scale) between macro- and micronutrients and fasting status altered the results in part, suggesting effect modification by fasting status (Table 3). For all types of acylcarnitines, hexose, sphingomyelins and lyso-phosphatidylcholines at least one interaction term was significant. The greatest influence of fasting time before blood sampling was seen for acylcarnitines. In the subgroup of acylcarnitines, each beta-estimator for fasting status was highly significant and the interaction terms protein*ft and mineral nutrients*ft for medium-chain acylcarnitines and dietary fiber*ft for long-chain acylcarnitines showed also significant *p*-values at a significance level of *p* < 0.1. Dietary fiber and protein showed a positive association with hexose and carbohydrates were negatively correlated. These associations were altered, when considering the corresponding interaction terms and changed the directions of the relationship. Fasting status also influenced concentrations of sphingomyelins and lyso-phosphatidylcholines. Especially the effect of protein intake on sphingomyelins was affected by fasting time. Fasting time showed no relevant effect modification on the metabolite subgroups amino acids and saturated, mono- and polyunsaturated phosphatidylcholines.

**Table 3:** Detailed results of the Generalized Linear Model^a^ with interaction terms included – beta estimators for the association between macronutrients (n = 7) and metabolite groups (n = 11), significance level < 0.1.

	Indispensable amino acids (n = 7)			Dispensable amino acids (n = 7)			Short-chain acylcarnitines (n = 5)			Medium-chain acylcarnitines (n = 6)			Long-chain acylcarnitines (n = 8)			Hexose (n = 1)		
	Estimate	SE	*p*	Estimate	SE	*p*	Estimate	SE	*p*	Estimate	SE	*p*	Estimate	SE	*p*	Estimate	SE	*p*
Alcohol	−0.12	0.09	0.16	**−0.15**	**0.09**	**0.09**	0.05	0.08	0.52	−0.01	0.08	0.94	0.06	0.08	0.50	−0.04	0.09	0.61
Dietary fiber	0.21	0.16	0.19	0.17	0.16	0.31	−0.19	0.15	0.21	0.10	0.16	0.53	**−0.34**	**0.15**	**0.03**	**0.42**	**0.16**	**0.01**
Protein	0.28	0.26	0.27	0.20	0.26	0.43	0.25	0.24	0.32	−0.34	0.25	0.17	−0.11	0.24	0.65	**0.63**	**0.25**	**0.01**
Fats	−0.30	0.19	0.11	−0.19	0.19	0.34	−0.09	0.18	0.63	0.05	0.18	0.77	−0.06	0.18	0.74	−0.32	0.19	0.09
Carbohydrates	**−0.28**	**0.16**	**0.09**	−0.10	0.17	0.54	0.00	0.16	0.98	−0.06	0.16	0.71	0.22	0.15	0.15	**−0.37**	**0.16**	**0.03**
Mineral nutrients	0.17	0.28	0.54	0.06	0.29	0.82	0.06	0.27	0.83	0.36	0.27	0.18	0.35	0.26	0.18	−0.31	0.28	0.27
Organic acids	−0.08	0.10	0.44	−0.17	0.10	0.10	−0.10	0.10	0.29	**−0.16**	**0.10**	**0.09**	**−0.19**	**0.09**	**0.04**	−0.07	0.10	0.49
Fasting time (ft)	−0.06	0.06	0.33	−0.06	0.06	0.31	**0.46**	**0.06**	**<0.0001** ^*^	**0.18**	**0.06**	<0.0001*	**0.45**	**0.06**	<0.0001*	−0.09	0.06	0.12
Alcohol*ft	0.09	0.06	0.16	0.11	0.07	0.11	0.03	0.06	0.65	0.05	0.06	0.43	0.04	0.06	0.53	0.08	0.06	0.21
Dietary fiber*ft	−0.08	0.12	0.51	−0.06	0.12	0.60	0.13	0.11	0.25	−0.10	0.12	0.38	**0.20**	**0.11**	**0.08**	**−0.29**	**0.12**	**0.01**
Protein*ft	−0.17	0.20	0.40	−0.18	0.21	0.38	−0.18	0.19	0.36	**0.36**	**0.19**	**0.07**	0.08	0.19	0.69	**−0.41**	**0.20**	**0.04**
Fats*ft	0.12	0.14	0.40	0.07	0.15	0.65	−0.01	0.14	0.96	−0.08	0.14	0.56	0.04	0.14	0.78	0.15	0.14	0.28
Carbohydrates*ft	0.10	0.13	0.42	0.08	0.13	0.53	−0.04	0.12	0.76	0.15	0.12	0.21	−0.09	0.12	0.45	**0.23**	**0.13**	**0.07**
Mineral nutrients*ft	−0.02	0.22	0.92	−0.02	0.22	0.93	0.07	0.21	0.76	**−0.40**	**0.21**	**0.06**	−0.28	0.21	0.18	0.24	0.22	0.27
Organic acids*ft	0.01	0.07	0.86	0.11	0.08	0.16	−0.03	0.07	0.67	0.05	0.07	0.45	0.04	0.07	0.54	0.03	0.07	0.72
	**Saturated phosphatidylcholines (n = 12)**			**Monounsaturated phosphatidylcholines (n = 11)**			**Polyunsaturated phosphatidylcholines (n = 49)**			**Sphingomyelins (n = 19)**			**Lyso-phosphatidylcholines (n = 9)**					
	**Estimate**	**SE**	***p***	**Estimate**	**SE**	***p***	**Estimate**	**SE**	***p***	**Estimate**	**SE**	***p***	**Estimate**	**SE**	***p***			
Alcohol	0.07	0.09	0.43	**0.23**	**0.09**	**0.01**	0.08	0.09	0.38	−0.10	0.09	0.27	0.05	0.09	0.60			
Dietary fiber	−0.13	0.16	0.44	−0.07	0.16	0.66	0.13	0.16	0.42	−0.07	0.16	0.67	0.07	0.16	0.66			
Protein	−0.02	0.26	0.95	0.26	0.25	0.30	0.21	0.26	0.42	−0.35	0.26	0.17	0.13	0.25	0.62			
Fats	−0.05	0.19	0.80	−0.29	0.19	0.12	−0.11	0.19	0.57	0.10	0.19	0.59	−0.12	0.19	0.52			
Carbohydrates	−0.12	0.16	0.45	0.00	0.16	1.00	−0.17	0.17	0.30	0.17	0.16	0.30	0.20	0.16	0.22			
Mineral nutrients	0.26	0.28	0.36	0.06	0.28	0.82	−0.10	0.28	0.72	0.19	0.28	0.50	−0.18	0.28	0.52			
Organic acids	−0.08	0.10	0.45	−0.11	0.10	0.25	−0.02	0.10	0.81	−0.09	0.10	0.36	−0.10	0.10	0.33			
Fasting time (ft)	0.07	0.06	0.22	0.05	0.06	0.41	0.02	0.06	0.75	**0.12**	**0.06**	**0.04**	**0.18**	**0.06**	<0.0001*			
Alcohol*ft	0.05	0.06	0.45	−0.02	0.06	0.75	0.05	0.07	0.44	0.05	0.06	0.42	0.02	0.06	0.76			
Dietary fiber*ft	0.08	0.12	0.48	−0.06	0.12	0.62	−0.09	0.12	0.45	0.02	0.12	0.86	−0.05	0.12	0.67			
Protein*ft	−0.03	0.20	0.89	−0.28	0.20	0.17	−0.16	0.20	0.44	**0.36**	**0.20**	**0.08**	−0.07	0.20	0.74			
Fats*ft	0.04	0.14	0.78	0.19	0.14	0.18	0.05	0.15	0.75	−0.08	0.14	0.60	0.06	0.14	0.67			
Carbohydrates*ft	0.05	0.13	0.70	0.07	0.13	0.57	0.12	0.13	0.36	−0.09	0.13	0.48	−0.15	0.13	0.23			
Mineral nutrients*ft	−0.10	0.22	0.64	0.08	0.22	0.71	0.13	0.22	0.57	−0.20	0.22	0.37	0.18	0.22	0.40			
Organic acids*ft	−0.01	0.07	0.94	0.01	0.07	0.93	−0.06	0.08	0.41	0.04	0.07	0.57	−0.01	0.07	0.88			

*p < 0.004 (0.05/11; Bonferroni-corrected).

^a^Adjusted for sex, age, BMI, smoked pack-cigarettes per year and physical activity.

**Figure 2:**
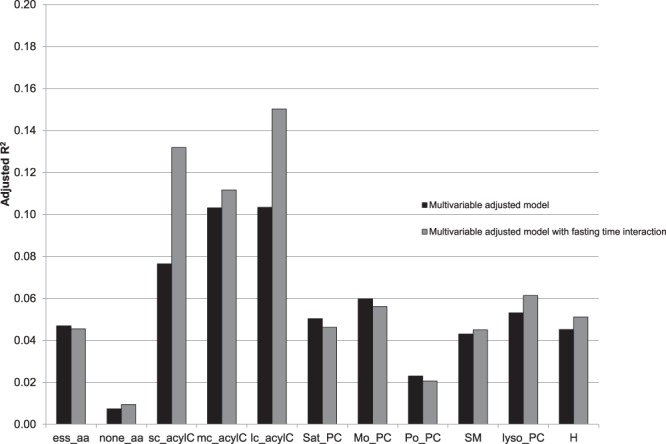
Explained variation of metabolites by macro- and micronutrients: adjusted R^2^-values comparing the multivariable adjusted models without and with interaction terms for fasting time included. The multivariable model was adjusted for sex, age, BMI, smoked pack-cigarettes per year and physical activity. ess_aa=indispensable amino acids; none_aa=dispensable amino acids; sc_acylC=short-chain acylcarnitines; mc_acylC=medium-chain acylcarnitines; lc_acylC=long-chain acylcarnitines; sat_PC=saturated phosphatidylcholines; mo_PC=monounsaturated phosphatidylcholines, po_PC= polyunsaturated phosphatidylcholines; SM=Sphingolipids; lysoPC=lyso-phosphatidylcholine and H=Hexose.

Figure 2 compares the adjusted R^2^ for models without and with including interaction terms for fasting time. The results showed only marginal changes with the highest increase in the proportion of explained variation for acylcarnitines, reflecting the influence of fasting time.

## Discussion

In a cross-sectional cohort study with 1197 middle-aged participants, we showed that self-reported macro- and micronutrients were associated with metabolite groups but had a very low prediction level. In the multivariable adjusted model, the proportion of explained variation (adjusted R^2^) by macro- and micronutrients ranged between 1% for dispensable amino acids and 10% for both medium- and long-chain PC (Fig. 2). These results are consistent with previous statements from the EPIC study^9^. Floegel *et al*. concluded that diet had only a minor association with the whole metabolic profile (proportion of explained variation ranged between 2% for hexose and amino acids and 6% for PC). The study design, a typical epidemiological investigation, is informative for the interpretation and design of cohort studies with non-fasting blood.

Two macronutrients were associated with most of the metabolite subgroups. Higher alcohol consumption and lower ingestion of organic acids were associated with higher levels of AcylC and PC. These results have been shown in several other studies. Krähenbühl showed increased levels of carnitines (long- and short-chain) in alcoholics compared to non-alcoholics^23^. Further, in a recent epidemiological study, alcohol consumption was associated with higher levels of monounsaturated PC^24^. Organic acids are produced from the catabolism of amino acids and are intermediates in metabolic pathways^25^. The association of self-reported amount of “organic acids” ingested and acylcarnitines is not unexpected, as the acylcarnitines are intermediate metabolites from organic acid metabolism^26^.

Our results suggested that saturated, mono- and polyunsaturated PC and amino acids are very stable biomarkers that seem unaffected by fasting status in a nutritional metabolomics setting. It has been known for years that only a small amount (1 to 6%) of the total free amino acid pool can be found in serum or plasma^27,28^. Thus, the metabolomic profile of serum amino acids has been used as nutritional marker very rarely^29^ but has an established, recognized utility as an indicator of nutritional deficiency states (i.e. limiting amino acid)^30,31^. Animal experiments in the 1960s have shown that plasma amino acid concentrations returned to a post-absorption steady state (reflective of the free total amino acid pool status) after 8 to 16 hours for dispensable and non-dispensable amino acid^31^. An example of the direct response of protein metabolism to carbohydrate intake is the reduction in plasma amino acid level (12% in the first hour), which occurs after the administration of glucose to fasting subjects^32^. We obtained similar results in our study, as shown in Tables 2 and 3, where carbohydrates had an independent negative effect, especially on indispensable amino acids, regardless of fasting status. More recent studies presented results similar to those reported in this paper; the reproducibility of amino acids was not particularly influenced by fasting status, possibly reflecting the genetic regulation of amino acid homeostasis^33,34^. However, medium- and long-chain AcylC, SM and Hexose showed nominal moderator effect of fasting on the associations between macro- and micronutrients and metabolites. These findings are also consistent with previous studies^33,34^. Carayol *et al*. measured the reproducibility of 16 acylcarnitine compounds and found out that they were particularly affected by fasting status. The authors suggested that this could be due to the fact that fatty acid oxidation is dynamically controlled by fasting time. Thus, if nutritional metabolomics studies in an epidemiological setting collecting non-fasted blood, fail to control for fasting time, results are expected to be partially biased. To our knowledge, this was the first study that examined the impact of fasting status on metabolite levels in a nutritional setting. Our results suggest that these metabolite subgroups can be reliably measured regardless of sample processing delay allowing flexibility to blood sample processing protocols of future studies measuring lipids and amino acids.

Participants of the CARLA cohort had fasted on average 200 minutes. Thus most of them cannot be classified as fasted, although it has been previously shown that the effect of food intake on the metabolic profile is already eliminated after 6 to 8 hours^12^. Thereby, we have with a median fasting time of 6.5 hours in the investigated subcohort, an acceptable range for fasted participants. It is generally accepted that the effect of diet on the metabolic profile in fasted participants with 12 or more hours of fasting, should be even greater. However, the requirement of 12 hours fasting time could have probably reduced considerably the participation willingness in the CARLA study. A further consideration deals with the fact that the metabolite concentrations changes rapidly in blood and not so fast in urine. Therefore urine probes are probably more suitable for nutritional metabolomics^12^. However, because of the high costs and very specialized technical competence associated with this technique, metabolomics is unlikely to be appropriate for screening of very large populations or for the routine clinical practice. And consequently ascertains the need to develop lower-cost procedures.

Metabolites determined in blood after a meal can be greatly altered. Also the digestion and resorption of metabolites can confound the association between nutrition and metabolomics^12,35^. Further factors influencing the concentrations of metabolites in blood, such as seasonal or circadian factors, or hormonal changes need to be considered. In order to investigate the effect of a dietary pattern on the metabolomics profile is recommended to standardise the blood withdrawal and choose only fasted participants (or at least correct for fasting time). A further strength of this study was the use of the good validated food frequency questionnaire from the EPIC cohort^18,19^. This questionnaire evaluates the nutritional pattern over a year and thus represents an average nutritional pattern. However, we also need to accept that, as with every questionnaire, there is an associated information bias. On the one hand, a “healthy” nutrition is socially desirable, and thus, the consumption of “unhealthy” ingredients is probably underestimated. On the other hand, a period of a year needs to be challenged, as the recollection ability can be impaired in such a long period. Finally, the population used in this study are older adults with a median age of 63 for men and 62 for women. The reported nutritional patterns reflect eventually, only the practices in mid-life. In order to generalise the results, younger adults and children need to be considered. The CARLA cohort is also known because of the high proportion of cardiovascular diseases and risk factors (74% of the men and 67% of the women are hypertensive)^36^. Therefore, the nutritional patterns could be distorted due to the cardiovascular profiles and be somewhat different in a healthy population.

## Conclusion

In summary, nutrition has an effect on metabolic products and can influence disease states. Particular dietary patterns play a role in the development and prevention of chronic diseases. It is also important to standardise the sampling of blood examinations to produce meaningful results. In this respect, fasting status is particularly relevant.

## Electronic supplementary material

Supplementary Information

## Data Availability

Data are subject to ethical and national data protection laws and are available only through an individual project agreement with CARLA. Requests should be sent to alexander.kluttig(at)medizin.uni-halle.de and are subject to approval by the CARLA Steering Committee (http://www.medizin.uni-halle.de/index.php?id=1109).
