# Supplementary material for: The human metabolic profile reflects macro- and micronutrient intake distinctly according to fasting time
Source: Sci Rep. 2018 Aug 16;8:12262. doi: 10.1038/s41598-018-30764-4 (PMC6095860; doi:10.1038/s41598-018-30764-4)
Supplement: Supplementary file 1 — Supplementary Information [file 41598_2018_30764_MOESM1_ESM.pdf]

The human metabolic profile reflects macro- and micronutrient intake distinctly according to fasting time

Sedlmeier A[1,2], Kluttig A[1], Giegling I[3], Prehn C[4], Adamski J[4,5,6], Kastenmüller G[6,7,8], Lacruz ME[1*]

Supplementary table 1: Mean and SD for metabolites in the study population in µmol/l (n=1197)

	Men (n=659)		Women (n=538)	
**Variable**	**Mean**	**SD**	**Mean**	**SD**
C0	48.64	13.52	42.66	10.33
C2	8.16	3.83	8.30	3.67
C3	0.49	0.20	0.40	0.15
C3-DC (C4-OH)	0.08	0.06	0.07	0.03
C4	0.26	0.11	0.23	0.11
C5	0.18	0.06	0.15	0.05
C5-OH (C3-DC-M)	0.04	0.01	0.04	0.01
C5:1	0.04	0.01	0.04	0.01
C6 (C4:1-DC)	0.09	0.03	0.09	0.03
C7-DC	0.04	0.02	0.03	0.01
C8	0.15	0.06	0.15	0.06
C8:1	0.15	0.08	0.14	0.07
C9	0.05	0.03	0.04	0.02
C10	0.25	0.11	0.24	0.10
C10:1	0.14	0.05	0.13	0.04
C10:2	0.05	0.02	0.05	0.01
C12	0.12	0.05	0.11	0.04
C12:1	0.11	0.04	0.11	0.04
C14:1-OH	0.01	0.00	0.01	0.00
C14:2	0.02	0.01	0.02	0.01
C16	0.14	0.05	0.12	0.04
C16:1-OH	0.01	0.00	0.01	0.00
C16:2	0.01	0.00	0.01	0.00
C16:2-OH	0.01	0.00	0.01	0.00
C18	0.06	0.02	0.05	0.02
C18:1	0.14	0.05	0.13	0.05
C18:1-OH	0.01	0.00	0.01	0.00
C18:2	0.05	0.02	0.04	0.01

Arg	122.93	22.06	123.05	22.71
Gln	597.11	104.14	580.51	108.24
Gly	242.85	56.21	287.99	101.35
His	84.22	27.00	81.02	16.33
Met	32.64	8.11	31.09	12.36
Orn	70.82	19.48	67.79	18.55
Phe	55.51	11.40	54.40	10.83
Pro	246.92	80.30	216.31	68.74
Ser	107.86	25.30	116.68	27.60
Thr	110.84	30.46	109.15	30.19
Trp	88.98	14.19	85.24	12.89
Tyr	102.29	28.10	100.73	29.55
Val	201.30	66.25	183.80	65.91
xLeu	239.36	68.19	214.44	61.37
lysoPC a C14:0	4.15	0.83	4.19	0.83
lysoPC a C16:0	91.29	25.60	84.55	23.91
lysoPC a C16:1	3.00	1.30	3.01	1.11
lysoPC a C17:0	1.47	0.54	1.57	0.59
lysoPC a C18:0	25.28	7.29	24.37	7.24
lysoPC a C18:1	20.70	7.43	19.15	6.30
lysoPC a C18:2	36.63	14.20	33.31	12.98
lysoPC a C20:3	2.58	0.99	2.48	0.88
lysoPC a C20:4	6.56	2.39	5.95	2.03
PC aa C26:0	0.76	0.22	0.78	0.24
PC aa C28:1	3.40	0.92	4.05	1.11
PC aa C30:0	5.98	2.14	6.65	2.28
PC aa C32:0	15.17	4.02	15.35	4.01
PC aa C32:1	20.29	12.87	21.00	10.91
PC aa C32:2	5.12	1.82	6.01	2.19
PC aa C32:3	0.57	0.18	0.70	0.23
PC aa C34:1	245.72	76.81	245.23	70.29
PC aa C34:2	406.51	112.67	411.42	119.16

PC aa C34:3	19.54	6.62	21.62	6.62
PC aa C34:4	2.46	0.95	2.80	1.07
PC aa C36:0	3.21	0.99	3.46	1.10
PC aa C36:1	59.69	18.26	62.61	16.64
PC aa C36:2	260.03	66.94	271.65	75.07
PC aa C36:3	158.72	41.55	168.65	45.45
PC aa C36:4	217.47	63.82	222.80	62.96
PC aa C36:5	39.21	20.76	39.49	20.17
PC aa C36:6	1.43	0.61	1.68	0.74
PC aa C38:0	3.19	1.00	3.49	1.18
PC aa C38:3	59.88	17.17	66.19	17.34
PC aa C38:4	122.00	36.98	129.41	35.65
PC aa C38:5	64.04	20.06	67.15	19.00
PC aa C38:6	104.31	33.81	110.90	36.10
PC aa C40:1	0.40	0.10	0.41	0.10
PC aa C40:2	0.38	0.12	0.39	0.13
PC aa C40:3	0.68	0.21	0.69	0.22
PC aa C40:4	4.10	1.50	4.15	1.19
PC aa C40:5	13.95	4.93	14.18	4.20
PC aa C40:6	37.21	13.47	39.95	14.03
PC aa C42:0	0.57	0.18	0.64	0.22
PC aa C42:1	0.28	0.08	0.31	0.10
PC aa C42:2	0.28	0.09	0.28	0.09
PC aa C42:4	0.19	0.05	0.19	0.05
PC aa C42:5	0.48	0.18	0.49	0.16
PC aa C42:6	0.60	0.19	0.65	0.19
PC ae C30:0	0.40	0.13	0.46	0.16
PC ae C30:2	0.17	0.06	0.21	0.07
PC ae C32:1	2.76	0.70	2.99	0.77
PC ae C32:2	0.67	0.18	0.81	0.23
PC ae C34:0	1.71	0.54	1.91	0.63
PC ae C34:1	10.11	2.63	11.49	3.08

PC ae C34:2	11.88	3.50	13.37	4.04
PC ae C34:3	7.82	2.38	8.81	2.87
PC ae C36:0	1.00	0.31	1.02	0.31
PC ae C36:1	8.13	2.21	9.44	2.62
PC ae C36:2	15.33	4.22	18.04	5.11
PC ae C36:3	8.95	2.52	9.93	2.85
PC ae C36:4	19.43	6.25	19.58	5.83
PC ae C36:5	13.68	4.10	13.92	4.27
PC ae C38:0	2.28	0.80	2.57	0.92
PC ae C38:2	2.27	0.57	2.56	0.66
PC ae C38:3	4.37	1.11	5.19	1.32
PC ae C38:4	13.52	3.45	14.62	3.74
PC ae C38:5	19.99	5.15	20.42	5.17
PC ae C38:6	8.41	2.41	9.11	2.67
PC ae C40:0	6.03	1.71	6.64	2.03
PC ae C40:1	1.44	0.41	1.48	0.43
PC ae C40:2	2.10	0.58	2.39	0.65
PC ae C40:3	1.04	0.23	1.22	0.27
PC ae C40:4	2.51	0.60	2.68	0.64
PC ae C40:5	4.02	0.92	4.29	1.05
PC ae C40:6	5.30	1.47	6.01	1.85
PC ae C42:0	0.49	0.10	0.50	0.11
PC ae C42:1	0.43	0.12	0.45	0.11
PC ae C42:2	0.66	0.17	0.72	0.21
PC ae C42:3	0.77	0.19	0.84	0.23
PC ae C42:4	0.96	0.26	1.02	0.27
PC ae C42:5	2.21	0.55	2.37	0.58
PC ae C44:3	0.13	0.04	0.14	0.04
PC ae C44:4	0.41	0.12	0.44	0.12
PC ae C44:5	1.91	0.60	2.00	0.61
PC ae C44:6	1.25	0.39	1.33	0.42
SM (OH) C14:1	7.53	2.70	9.25	3.09

SM C16:0	125.79	34.97	136.17	34.94
SM C16:1	19.51	5.59	23.57	6.30
SM (OH) C16:1	4.07	1.43	5.06	1.65
SM C18:0	31.67	9.23	36.58	10.16
SM C18:1	14.19	4.47	18.18	5.40
SM (OH) C22:1	16.60	5.94	19.66	6.39
SM (OH) C22:2	13.44	4.75	17.51	5.70
SM C24:0	29.17	9.88	30.13	9.08
SM C24:1	71.97	21.83	75.31	21.13
H1	6411.43	1441.48	6032.16	1125.89

Supplementary table 2: Micro- and macronutrient intake (in g/day) for the study population (n=1197)

	**Men (n=659)**		**Women (n=538)**	
	**Mean**	**SD**	**Mean**	**SD**
Alcohol	19.95	21.62	7.19	10.22
Dietary fiber	25.46	7.90	21.91	6.36
Protein	91.04	24.36	65.07	18.32
Fats	111.60	31.83	78.77	22.70
Carbohydrates	272.33	82.80	215.00	69.80
Mineral nutrients	19.38	4.61	15.21	3.72
Organic acids	7.28	3.19	7.03	3.71
